# Gastrotomy for Incised Wound of Stomach

**Published:** 1887-12

**Authors:** J. A. Black

**Affiliations:** Round Rock, Tevas


### GASTROTOMY; FOR INCISED WOUND OF STOMACH; FISH BONES REMOVED FROM THE ABDOMINAL CAVITY.

Read beforeAustin District Medical Association, and voted to be publshed in Daniel's Texas Medical Journal.

*By J. A. Black, M. D., Round Rock, Tevas.*

JULY 20, 1887, at 9 o'clock p. m., Wm. Edwards, farmer, white, age 17, married, was cut while attending a country dance. The long blade of a large pocket knife entered the abdomen at the left side, immediately below the lower margin of ribs, about five inches from the median line, making an external wound two inches long, extending toward the median line, parallel with the margin of ribs, from which escaped undigested food, and blood.

July 21, at 9 o'clock a. m., 12 hours from the time of the injury, I saw the patient for the first time. He had tossed about all night, in great pain, and without medical attention. I gave anodynes and placed him in a position which favored the escape of the food and blood until assistance could arrive. Four hours later, Dr. T. J. Bennett, of Austin, with my assistance, proceeded to operate by enlarging the original cut in the direction of the median line,. First an incision about two inches in length was made, which proving to be inadequate, a further incision was carried to the median line, making a total external opening of five and a half inches transversely across the abdomen, terminating just below the xyphoid cartilage. Several large fish bones, some of them over an inch in length, and a quarter of an inch in diameter, together with a quantity of undigested food, and some blood, were found lying on the great omentum and intestines, and were removed. The small intestines, as well as the transverse and a portion of the descending colon, were pulled outside, laid upon aseptic towels, and searched for wounds, but none were found. The investigation was extended above the line of incision, when it was found that the blade had passed through the great omentum and entered the stomach near the center of the great curvature, making a wound about one and a half inches in length.

The stomach was then drawn outside, and two interrupted and two continuous sutures, making four in all, were made in the wound; fine iron-dyed silk was used to close the wound. Catgut was thought of, but it was feared that the gastric juices would digest the sutures before union could take place. As the stomach was pulled outside vomiting took place, and some depression was noticeable for a short time. It is worthy of note, that, while vomiting was taking place, the stomach was held in the hand of the operator, and the firm and uniform contractions which the muscular structures would indicate to take place in vomiting, were actually felt. Absorbent cotton was used instead of sponges. The viscera were carefully cleansed and returned, and the abdominal cavity mopped out with the cotton. No irrigation was employed. The external wound was then closed by eight interrupted sutures of strong iron-dyed silk, the peritoneum being included in each one. Space was left for the escape of pus, in case any should form.

Iodoform and carbolated absorbent cotton, confined by a broad bandage, tightly applied, constituted the dressing. The patient was placed on his back, with the shoulders elevated, and a roll of quilt placed under the knees. Anodyne and nutrient enemas—only ice water to enter the stomach—position of patient- attention to bladder, etc., constituted the chief directions given the nurse after operation.

July 22, temperature 102. Vomiting at short intervals since the operation, until about noon to-day—23 hours. Some trouble in urinating, relieved by hot cloths over lower part of abdomen. Severe headache all the time. Some swelling—no discharge from wound. Treatment continued.

Jaly 23. Temperature 101, pulse 103. Nausea, no vomiting. Still severe headache. Wound in apparently same condition as yesterday. Treatment about the same.

July 24. Some discharge from wound—a little nausea occasionally; headache. Bowels moved by simple enema. Temperature and pulse about same.

July 25, fourth day. Some swelling about wound; general tympanites; headache. Good deal of pus discharging from lower edge of wound; also, some from suture points; sutures were taken out and rubber adhesive plaster applied. First nourishment by mouth; boiled milk, lime water iced, turpentine stupes over abdomen.

July 26 and 27. Less swelling, free discharge of pus. Temperature normal, pulse weak, pains over abdomen, not so much headache. Gave beef tea, soft boiled eggs, boiled milk: opium for pain.

July 28. Temperature normal, pulse weak, rapid, somewhat irregular, violent diarrhœa, patient somewhat despondent. Brandy, boiled milk, morphia, plumbi acetas, *pro re nota.*

July 29. Better. No diarrhœa, very little discharge of pus. Diet and treatment continued.

July 30. Pulse good, temperature normal. Bowels have not moved since evening of 28; abdomen soft, very little soreness. Stimulating nourishment continued.

August 1. Afternoon. Patient was attacked with severe colliquative diarrhœa, probably eliminative. Patient nearly washed into the great unknown. Stools offensive, no pain. Gallic acid, ergot, opium. Nourishment with pepsin.

External wound nearly healed August 3.

Diarrhœa continued with more or less severity until August 6.

Bowels did not move any more until August 10.

Good nourishment kept up and relished; digestive tonics. Patient went on to perfect recovery. [Patient was present at the meeting. Ed.]

